# Recent Advances in Bacteria Identification by Matrix-Assisted Laser Desorption/Ionization Mass Spectrometry Using Nanomaterials as Affinity Probes

**DOI:** 10.3390/ijms15057266

**Published:** 2014-04-28

**Authors:** Tai-Chia Chiu

**Affiliations:** Department of Applied Science, National Taitung University, 684 Section 1, Chunghua Road, Taitung 95002, Taiwan; E-Mail: tcchiu@nttu.edu.tw; Tel.: +886-89-353840; Fax: +886-89-342539

**Keywords:** affinity probes, bacteria, matrix-assisted laser desorption/ionization mass spectrometry, nanomaterials

## Abstract

Identifying trace amounts of bacteria rapidly, accurately, selectively, and with high sensitivity is important to ensuring the safety of food and diagnosing infectious bacterial diseases. Microbial diseases constitute the major cause of death in many developing and developed countries of the world. The early detection of pathogenic bacteria is crucial in preventing, treating, and containing the spread of infections, and there is an urgent requirement for sensitive, specific, and accurate diagnostic tests. Matrix-assisted laser desorption/ionization mass spectrometry (MALDI-MS) is an extremely selective and sensitive analytical tool that can be used to characterize different species of pathogenic bacteria. Various functionalized or unmodified nanomaterials can be used as affinity probes to capture and concentrate microorganisms. Recent developments in bacterial detection using nanomaterials-assisted MALDI-MS approaches are highlighted in this article. A comprehensive table listing MALDI-MS approaches for identifying pathogenic bacteria, categorized by the nanomaterials used, is provided.

## 1. Introduction

Worldwide, infectious diseases cause nearly 40% of the total 50 million deaths annually [1]. According to the World Health Organization, microbial hazards are the primary concern [2] because microbial diseases are a major cause of death in many developing and developed countries of the world [3,4]. Therefore, the development of rapid, accurate, and sensitive methods for bacterial identification is important for the clinical diagnosis, efficient treatment and prevention of diseases, environmental monitoring and food safety [5–8]. In clinical laboratories, bacterial identification is typically based on phenotypic tests, including Gram staining, culture and growth characteristics, and biochemical patterns. A number of methods are currently employed to detect and identify pathogenic agents, and these mainly rely on specific microbiological and biochemical identification methods [9–11]. These methods include culturing the microbes and counting the bacterial colonies, immunology-based methods, antigen–antibody interaction methods, and the polymerase chain reaction method, which involves DNA analysis. These methods can be sensitive and inexpensive, and can provide both qualitative and quantitative information about the test microorganisms; however, they are often time-consuming and laborious because each involves a pathogen amplification step. At present, most bacteria can be identified between a few hours to 1–2 days using these methods, with slow-growing microorganisms requiring additional time or supplementary tests [12]. Consequently, there is an urgent requirement for developing a rapid, sensitive, and selective detection method for such pathogens to treat individuals at risk, to improve public health surveillance and epidemiology, which is essential for ensuring the safety of food supplies, and to diagnose infectious bacterial diseases accurately.

There are challenges associated with identifying various types of pathogenic bacteria in a wide range of samples. Matrix-assisted laser desorption/ionization mass spectrometry (MALDI-MS) has been used to analyze various biomolecules, including peptides, proteins, DNA, RNA, oligonucleotides, oligosaccharides, and polymers [13–16]. This approach was first introduced by Tanaka and Karas in the late 1980s [17,18], and is a soft ionization method that provides mass spectra of the analytes with a minimum amount of fragmentation. MALDI-MS has a number of advantages over conventional methods including ease of operation, providing structural information of molecules with high throughput, speed, sensitivity, accuracy, and reproducibility [19,20]. Therefore, it has become a powerful tool for rapid characterization, differentiation, and identification of microorganism species [21–27]. For example, the mass-spectral profiling of whole cells can indicate the presence of unique biomarkers that can serve as the basis for identifying microbes [28,29]. In general, mass spectra of microbes isolated from a sample may contain unique patterns that can be automatically matched with spectra in a well-established reference library of microorganisms that have been characterized using appropriate sample preparation protocols. Matching the spectra allow the microbes to be identified as well as evaluated.

A sufficient number of bacterial cells (typically ~10^4^ cells per well) are required to generate detectable MALDI-MS ion signals. However, samples obtained from infectious biological fluids or food poisoning samples are difficult to characterize directly by MALDI-MS because the ions generated from the bacterial cells may be seriously suppressed by the complex sample matrices. This led to the idea of using nanoparticles as affinity probes, to enhance the ability of MALDI-MS to detect bacteria [30,31]. Nanoparticles provide a high surface to volume ratio, giving them high binding and capture efficiencies for bacteria. Affinity separation approaches are methods of selectively concentrating trace amounts of bacteria from complex biological and food samples before they are characterized using MALDI-MS. When inorganic nanoparticles are used in MALDI-MS, instead of organic matrices, the method is called surface-assisted laser desorption and ionization MS (SALDI-MS) [32–36]. SALDI-MS was originally proposed by Sunner and Chen as early as 1995, and graphite particles were originally used as ion emitters [37]. This method was called SALDI-MS to emphasize that the surfaces and surface structures are critical to not only sample preparation but also desorption and ionization processes [38]. Numerous types of nanoparticles such as gold (Au) nanoparticles [39–41], silver (Ag) nanoparticles [42,43], magnetic nanoparticles [44,45], titanium dioxide (TiO_2_) nanoparticles [46,47], carbon nanotubes [48,49], carbon nanoparticles [50], nanodiamonds [51], and graphene and graphene oxide [52] have been successfully used as matrices in SALDI-MS. The nanomaterials used in SALDI-MS play similar roles to the organic matrices used in MALDI-MS, absorbing energy from the laser irradiating them and efficiently transferring the energy to the analytes, causing the analytes to be desorbed and ionized [30]. The method provides several advantages including lower background noise in the low mass region, high surface areas, simple sample preparation, flexibility in sample desorption under different conditions, and high UV absorptivity [34]. Nanoparticles can also act as affinity probes, making it easy to concentrate the analytes, and offering good sensitivity and reproducibility [34].

In this review article, we focused on the overview of the recent advancements in the use of nanoparticles as affinity probes to enhance the detection sensitivity and selectivity of bacteria using MALDI-MS. Several examples of successful MALDI-MS approaches for detecting pathogenic bacteria have been provided to illustrate the advantages of this approach with respect to simplicity, sensitivity, and reproducibility. Furthermore, this article also provides some examples for the identification of bacteria in real samples using nanomaterials-assisted MALDI-MS approaches.

## 2. Bacterial Identification Using MALDI-MS

MALDI-MS is a very sensitive method where a single bacteria colony is sufficient for analysis, while other methods typically require culturing or enrichment of bacteria to obtain sufficient materials. Therefore, in clinical microbiological laboratories, the MALDI-MS is increasingly used for bacterial identification through the determination of the exact molecular masses of numerous peptides and small proteins, many of which are ribosomal. Conventional biochemical differentiation methods [24] have already been replaced by MALDI-MS. Because MALDI-MS is primarily applicable for analyzing clonal isolates, cultivation of the microorganism is still required. Moreover, for accurate identification, MALDI-MS can be used directly on the clinical samples that contain very few bacteria for accurate identification. In 2010, Ferreira *et al.* [53] introduced a MALDI-MS method for direct analysis of urine samples (4 mL) and observed that the inoculum level in the samples must be greater than 10^5^ cfu/mL (colony-forming unit/mL). In 2010, a protocol for direct analysis of blood was introduced by Stevenson *et al.* [54], who separate bacteria from the red blood cells and plasma proteins via several centrifugation steps. A total of 212 positive cultures representing 32 genera and 60 species or groups were examined. Besides urine and blood, Barreiro *et al.* [55] inoculated pasteurized and homogenized samples of whole milk with the bacterial loads of 10^3^–10^8^ cfu. Sepsityper™ Kit (Bruker, Billerica, MA, USA) was used to for the testing milk sample and then analyzed by the Bruker BioTyper database. For a slightly contaminated (10^4^ cfu/mL bacteria) milk sample, bacterial identification could be performed after initial incubation at 37 °C for 4 h. The detection limits for bacteria were in the range of 10^6^–10^7^ cfu/mL.

## 3. Nanoparticles Used as Affinity Probes

Nanoparticles are clusters of a few hundred to a few thousand atoms, and range from 1 to 100 nm in diameter. The chemical and physical properties of nanoparticles depend on their surfaces; therefore, these properties are highly dependent on the sizes, shapes, and compositions of the nanoparticles [56–58]. Nanoparticles have high surface-to-volume ratios, and those with excellent optical, magnetic, and electronic properties have been employed in sensing, imaging, catalysis, electronics, optics, and optoelectronics applications [59–65]. Nanoparticles can play an important role in determining the sensitivity of MALDI-MS and provide a high surface-to-volume ratio to give a high binding efficiency for bacteria. The affinity separation approach has been used to attempt to selectively concentrate trace amounts of bacteria from biological and food samples. Nanoparticles (functionalized or unmodified) that have been used as affinity probes to increase the sensitivity of MALDI-MS for detecting microbes are summarized in Table 1.

### 3.1. Magnetic Nanoparticles

**Figure 1.**
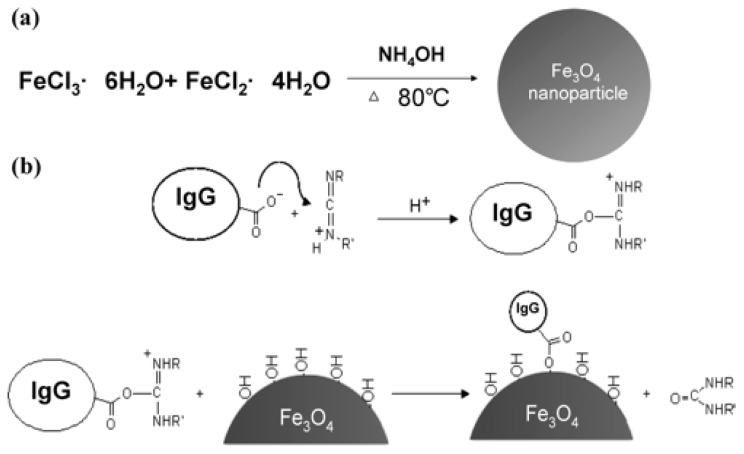
Synthetic route for immobilizing immunoglobulin (IgG) onto the surfaces of Fe_3_O_4_ magnetic nanoparticles. Reprinted with permission from [66]. Copyright (2014) American Chemical Society.

Ho *et al.* [66] immobilized human immunoglobulin (IgG) onto the surfaces of magnetic Fe_3_O_4_ nanoparticles through covalent bonding (Figure 1). The functionalized magnetic nanoparticles were used as affinity probes to selectively concentrate pathogens, such as *Staphylococcus aureus* (*S. aureus*) and *Staphylococcus saprophyticus* (*S. saprophyticus*), from sample solutions. The bacteria were then characterized using MALDI-MS. The lowest bacterial concentration detected in an aqueous sample solution (0.5 mL) was 3 × 10^5^ cfu/mL, for both *S. aureus* and *S. saprophyticus*, and the lowest detectable *S. saprophyticus* concentration in a urine sample was 3 × 10^7^ cfu/mL.

**Figure 2.**
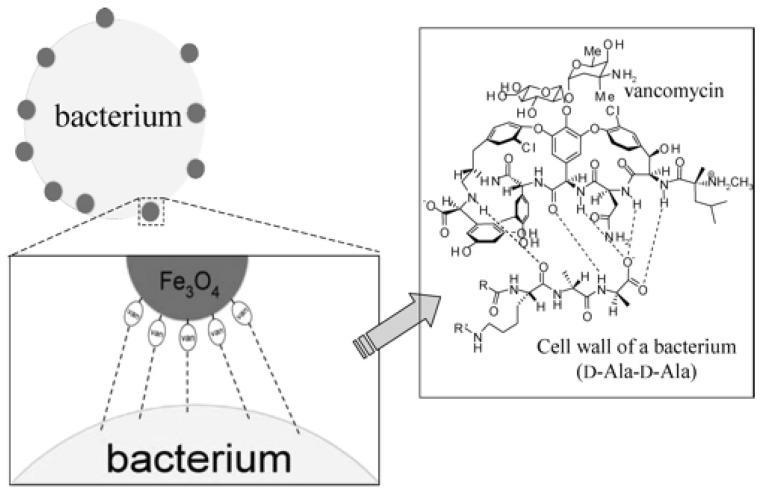
Cartoon illustrations of the proposed method for anchoring vancomycin-immobilized magnetic nanoparticles onto the surface of a Gram-positive bacterial cell and the binding of vancomycin to the terminal of *D*-Alanine (*D*-Ala)–*D*-Ala units of the peptides on the cell wall of a Gram-positive bacterium. Reprinted with permission from [67]. Copyright (2014) American Chemical Society.

Vancomycin-modified 11 nm magnetic (Fe_3_O_4_) nanoparticles were used as affinity probes to selectively bind to the surface walls of Gram-positive bacteria (*S. aureus* and *S. saprophyticus*), as shown in Figure 2, allowing the bacteria to then be directly characterized using MALDI-MS [67]. Vancomycin is one of the most potent antibiotics, and has a high specificity for the *D*-Alanine (Ala) (*D*-Ala) moieties on the cell walls of Gram-positive bacteria. The lowest cell concentrations that could be detected in a urine sample (3 mL) were 7.4 × 10^4^ cfu/mL for *S. aureus* and 7.8 × 10^4^ cfu/mL for *S. saprophyticus*.

IgG- and vancomycin-modified magnetic nanoparticles have been demonstrated to exhibit effective affinities for selectively concentrating traces of bacteria from the sample solutions. However, because interferences from the urine matrix affect the binding capacity of these nanoprobes, further improvements are required to reduce the matrix effects in the analysis of biological samples.

A combination of membrane filtration and vancomycin-modified magnetic (Fe_3_O_4_) 15–20 nm nanoparticles has been used to selectively concentrate Gram-positive bacteria from tap water and reservoir water, allowing the bacteria to be rapidly analyzed using whole-cell MALDI-MS [68]. The capture efficiency for Gram-positive bacteria using these vancomycin-modified magnetic nanoparticles was 26.7%–33.3%, and the analysis time was approximately 2 h. This approach enhanced the sensitivity of the method by a factor of approximately 6 × 10^4^, giving a limit of detection of 5 × 10^2^ cfu/mL for *Bacillus cereus* (*B. cereus*), *Enterococcus faecium* (*E. faecium*), and *S. aureus* in water samples (2 L).

### 3.2. Silver (Ag) Nanoparticles

The bifunctional properties of Ag nanoparticles allowed them to be used as affinity probes for *Escherichia coli* (*E. coli*) and *Serratia marcescens* (*S. marcescens*), by Gopal *et al.* [69], to increase the sensitivity of MALDI-MS when characterizing the bacteria. The critical concentration of affinity probes for Ag nanoparticles was 1 mL/L in the case of *E. coli* and 0.5 mL/L in the case of *S. marcescens*. Ag nanoparticle concentrations higher than these values showed pronounced bactericidal activities.

The same research group also observed that an ionic solution (CrO_4_^2−^) and 0.035 mg of Ag nanoparticles could be used to capture yogurt bacteria (*Bifidobacterium lactis* (*B. lactis*), *Lactobacillus acidophilus* (*L. acidophilus*), *Streptococcus thermophilus* (*S. thermophilus*), and *Lactobacillus bulgaricus* (*L. bulgaricus*) from AB yogurt and *L. acidophilus*, *Bifidobacterium longum* (*B. longum*), *L. bulgaricus*, and *S. thermophilus* from Lin yogurt), improving the sensitivity achieved for detecting bacteria in yogurt samples [70]. This method has demonstrated a rapid, selective and sensitive means of bacterial detection using MALDI-MS for food microbiology.

### 3.3. Cadmium Sulfide (CdS) Quantum Dots (QDs)

Gopal *et al.* [71] has reported that CdS QDs can degrade the extracellular polysaccharides of *E. coli* cells when using MALDI-MS. Adding 20 μL/L of CdS QDs was observed to enhance the extracellular polymeric substance (EPS) peaks using an incubation time of up to 3 h. The authors confirmed that CdS QDs can function as more than just affinity probes, being able to degrade EPSs. CdS QDs can, therefore, be used to inactivate pathogenic *E. coli* and also inhibit the growth of *E. coli* biofilms.

Manikandan and Wu [72] observed that CdS QDs (10 mg/L) with particle sizes of 1–7 nm performed fungicidal roles and functioned as protein signal enhancement probes in the MALDI-MS analysis of the fungi *Saccharomyces cerevisiae* (*S. cerevisiae*) and *Candida utilis* (*C. utilis*). From their MALDI-MS results, the authors proposed the mechanism involving the CdS QDs interacting with the EPSs and removing small molecules from the EPS layers. The MALDI-MS protein signals were enhanced at all of the CdS QD concentrations that were tested (10–30 mg).

Chitosan-modified CdS QDs have been used as effective bacterial biosensors because of the strong affinities between chitosan molecules and bacterial membranes [73]. In that study, *Pseudomonas aeruginosa* (*P. aeruginosa*) and *S. aureus* cells were detected at low concentrations, 200 and 150 cfu/mL, respectively, after an extremely short time (1 min). MALDI-MS and transmission electron microscopy were used to confirm the interactions and the biocompatibility of the chitosan-modified CdS QDs with bacterial cells.

### 3.4. Platinum (Pt) Nanoparticles

Ahmad and Wu [74] employed a single drop microextraction technique, using an ionic liquid (1-butyl-3-methylimidazolium hexafluorophosphate) drop mixed with Pt nanoparticles, to extract bacterial proteins from aqueous samples to characterize pathogenic bacteria using MALDI-MS. This approach is based on surface changes in the ionic liquid and the membrane proteins of the bacteria, and it was successfully used to detect *E. coli* and *S. marcescens* at concentrations as low as 10^6^ cfu/mL.

A rapid method for detecting bacteria associated with plants by an on-particle ionization and enrichment approach using IgG-functionalized Pt nanoparticle-assisted MALDI-MS was reported by Ahmad *et al.* [75]. The approach was successfully used to detect *Bacillus thuringiensis* (*B. thuringiensis*) and *Bacillus subtilis* (*B. subtilis*) isolated from rhizospheric soil and carrot plant roots. This study proved that bacteria can be directly detected even at low concentrations.

A rapid and sensitive approach to studying interactions between an affinity probe and a bacterial wall was introduced by Ahmad and Wu [76]. IgG was immobilized on Pt nanoparticles and MALDI-MS was used to detect the specific surface proteins of the bacteria *S. marcescens* and *E. coli*. This approach enabled the rapid detection of bacterial proteins, at a high resolution and with good sensitivity, without the need for tedious washing and separation procedures, and can be used to detect approximately 10^5^ cfu/mL of *S. marcescens* and *E. coli*.

### 3.5. Other Nanomaterials

**Figure 3.**
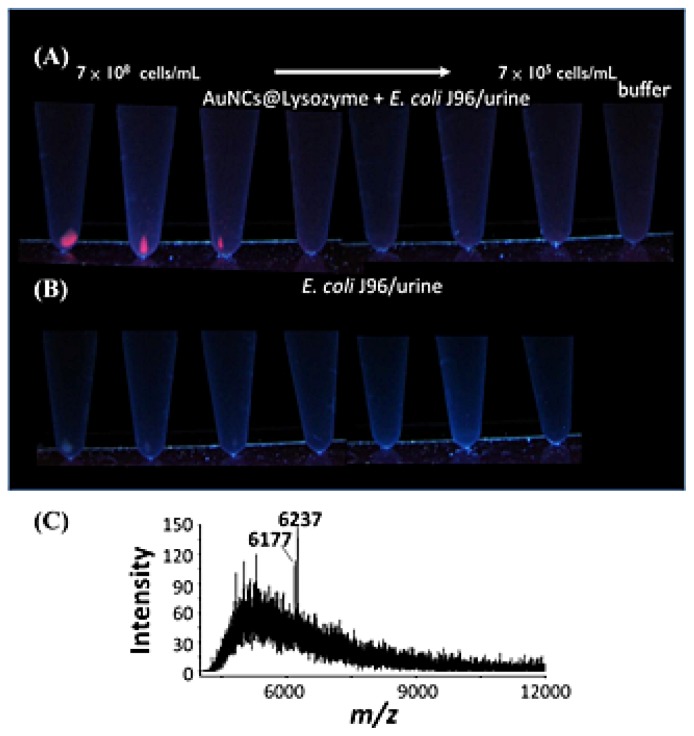
Photographs obtained by vortex-mixing (**A**) the lysozyme-AuNCs with *E. coli* J96 at different cell concentrations and (**B**) *E. coli* J96 alone for 1 h at different cell concentrations, followed by centrifugation at 3500 rpm for 5 min. The samples were prepared in urine that was diluted 50-fold with PBS solution (pH 7.4) containing BSA (~10 μM). The photographs were taken under illumination of UV light (λ_max_ = 365 nm); (**C**) Examination of the limit of detection. Matrix-assisted laser desorption/ionization mass spectrometry (MALDI-MS) obtained after using the lysozyme-AuNCs (1.36 mg/mL, 0.1 mL) as affinity probes to concentrate target species from a urine sample (0.90 mL) containing *E. coli* J96 (1.59 × 10^6^ cells/mL) for 1 h. The urine sample was diluted 50-fold with PBS solution (pH 7.4) containing BSA (~10 μM) prior to bacterial spiking. Reprinted with permission from [77].

Chan *et al.* [77] demonstrated that pathogenic bacteria, including *E. coli*, *Klebsiella pneumonia* (*K. pneumoniae*), *P. aeruginosa*, pandrug-resistant *Acinetobacter baumannii* (*A. baumannii*), *S. aureus*, *E. faecalis*, and vancomycin-resistant *E. faecalis*, can be concentrated by lysozyme-encapsulated gold nanoclusters (AuNCs) that photoluminesce red, and distinguished by the results combining MALDI-MS and principal component analysis. Figure 3A shows photographs of sample tubes after the lysozyme-AuNCs were used as probes for *E. coli* J96 in urine samples containing different concentrations of the *E. coli*. Photographs of the control experiment sample tubes are shown in Figure 3B for comparison. Figure 3C shows the MALDI-MS spectra of the conjugates containing *E. coli* J96, in which the peaks at *m*/*z* 6177 and 6237 correspond to *E. coli* J96. The lowest *E. coli* concentration that could be detected using this approach was approximately 10^6^ cfu/mL. The advantages of this method include speed (without cell culturing) and simplicity, and it can be used as universal affinity probes for Gram-positive/negative and antibiotic-resistant bacteria.

Abdelhamid and Wu [78] demonstrated that multifunctional graphene magnetic nanosheets modified with chitosan (GMCS) can be used in MALDI-MS for the sensitive detection of pathogenic bacteria (*P. aeruginosa* and *S. aureus*). The GMCS were observed to act as efficient separation and preconcentration nanoprobes for SALDI and enhance the ionization of bacterial biomolecules. GMCS have been used in the direct detection of low concentrations of *P. aeruginosa* and *S. aureus* in blood samples, demonstrating their practical applicability. This approach offers many advantages such as robustness, simplicity, and the capability for fluorescence based real-sample monitoring.

The heat stress response of *E. coli* (at 10^7^ cfu/mL) at different temperatures has been studied using nickel oxide (NiO) nanoparticle-assisted MALDI-MS by Hasan *et al.* [79]. MALDI-MS was successfully used to detect 10 kDa chaperonin proteins produced by *E. coli* under heat stress at temperatures between 40 and 80 °C in the absence or presence of NiO nanoparticles. Dramatic decreases in the viability of *E. coli* in the presence of NiO were confirmed from the MALDI-MS results. This technique is a rapid, sensitive, and efficient approach for bacterial detection under extremely harsh conditions.

Gopal *et al.* [80] demonstrated that *S. aureus* isolated from the human nasal passage can be directly detected using MALDI-MS assisted by TiO_2_ nanoparticles, without any culturing steps or sample pretreatment being required. TiO_2_ nanoparticles were used to enhance the bacterial signals in the direct MALDI-MS analysis.

MALDI-MS has been used to evaluate bactericidal activity, by detecting proteins produced because of the inactivation of *E. coli* cells by zinc oxide (ZnO) nanoparticles [81]. The results showed that at concentrations of 1 and 5 g/L ZnO nanoparticles can be used as affinity probes to improve the signal intensities in the MS spectra. The significant differences in the spectral patterns confirmed that MALDI-MS was successfully used to evaluate the bactericidal activity of ZnO nanoparticles.

**Figure 4.**
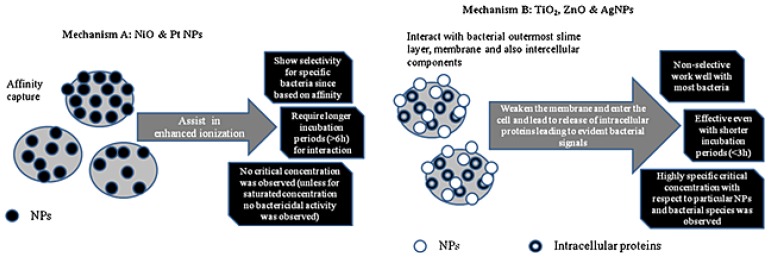
Schematic diagram showing the mechanisms (Mechanism A and Mechanism B) for interactions of five nanoparticles with two pathogenic bacteria postulated in the study. Reprinted with permission from [82].

Gopal *et al.* [82] proposed mechanisms for the interactions between five nanoparticles (Ag, NiO, Pt, TiO_2_, and ZnO) and two bacteria (*S. aureus* and *P. aeruginosa*) from studies using transmission electron microscopy, ultra spectrometry, and MALDI-MS. Two mechanisms (Figure 4) were proposed for the interactions: (1) Mechanism A was proposed for Pt and NiO nanoparticles, the function of which is based on their affinities for bacterial walls; and (2) Mechanism B was proposed for bactericidal nanoparticles, such as TiO_2_, ZnO, and Ag nanoparticles.

## 4. Conclusions

MALDI-MS is an emerging analytical tool for detecting and identifying microorganisms. It offers high sensitivity, simple sample preparation processes, low sample consumption volumes, and the possibility of automated and high-throughput analyses. In this review, we have described several MALDI-MS approaches for detecting pathogenic bacteria using nanomaterials (such as AuNCs, Ag, magnetic, and Pt nanoparticles and CdS QDs) as affinity probes. The nanomaterials described here act as concentration probes for the selective capture of unique biomarkers from microorganisms, and as surfaces to absorb energy from the laser irradiation, thereby inducing desorption and ionization of the analytes.

As mentioned above, the most important advantage of the affinity-based nanoparobe methods is their ability to selectively concentrate and purify microorganisms from complex samples, such as urine and blood, and allow the further identification of microorganisms without microbial culturing using MALDI-MS. For the nanomaterials-assisted MALDI-MS, Direct analysis of microorganisms at low microbial levels can be performed using the nanomaterials-based MALDI-MS. Numerous nanomaterials have been demonstrated to be useful as affinity probes for targeting bacteria. However, some nanoparticles such as Ag, TiO_2_ and ZnO, also exhibit bactericidal activity, and therefore might not be good affinity probes at higher nanoparticles concentrations. Controlling of the nanoparticle concentration will be a key factor. All these nanomaterials-assisted MALDI-MS methods also encounter challenges with respect to the enrichment of unknown target bacterial species from the urine, blood, and cerebrospinal fluid. Thus, a limiting factor in MALDI-MS analysis is insufficient database entries. The addition of certain species to the database has been demonstrate significantly improve MALDI-MS precision in bacterial identification.

The broad adoption of nanomaterials-assisted MALDI-MS methods for bacterial identification will require a substantial improvement in performance compared with the existing methods, such as conventional MALDI-MS and biochemical tests. Thus, the standardization of terminology is required. Advances in nanomaterials-assisted MALDI-MS methods will support the simple and accurate means of bacterial identification for food safety, environmental monitoring and clinical diagnosis.

## Figures and Tables

**Table 1.** Nanomaterials used as affinity probes in MALDI-MS.

Nanomaterials	Functionalized molecule	Pathogen	Application	LOD (cfu/mL)	Ref.
Fe_3_O_4_ NPs	IgG	*S. aureus; S. saprophyticus*		3.0 × 10^5^	[66]
Fe_3_O_4_ NPs	IgG	*S. saprophyticus*	Urine	3.0×10^7^	[66]
Fe_3_O_4_ NPs	Vancomycin	*S. aureus; S. saprophyticus*	Urine	7.8 × 10^4^; 7.4 × 10^4^	[67]
Fe_3_O_4_ NPs	Vancomycin	*B. cereus; E. faecium; S. aureus*	Tap water, reservoir water	5.0 × 10^2^	[68]
Ag NPs		*E. coli; S. marcescen*		N.D.	[69]
Ag NPs		*B. lactis; L. acidophilus; S. thermophilus; L. bulgaricus*	Yogurt	N.D.	[70]
Ag NPs		*L. acidophilus; B. longum; L. bulgaricus; S. thermophilus*	Yogurt	N.D.	[70]
CdS QDs		*E. coli*		N.D.	[71]
CdS QDs		*S. cerevisiae; C. utilis*		N.D.	[72]
CdS QDs	Chitosan	*P. aeruginosa; S. aureus*		2.0 × 10^2^; 1.5 × 10^2^	[73]
Pt NPs	Mixed with ionic liquid (1-butyl-3-methylimidazolium hexafluorophosphate)	*E. coli; S. marcescens*		10^6^	[74]
Pt NPs	IgG	*B. thuringiensis; B. subtilis*	Rhizospheric soil and root	N.D.	[75]
Pt NPs	IgG	*S. marcescens; E. coli*		10^5^	[76]
AuNCs	Lysozyme	*E. coli; K. pneumoniae; P. aeruginosa*; pandrug-resistant *A. baumannii; S. aureus; E. faecalis;* vancomycin-resistant *E. faecalis*	Fetal bovine serum; urine	N.D.; 10^6^	[77]
Graphene magnetic nanosheets	Chitosan	*P. aeruginosa; S. aureus*	Blood	6.0 × 10^2^; 5.0 × 10^2^	[78]
NiO NPs		*E. coli*		10^7^	[79]
TiO_2_ NPs		*S. aureus*	Human nasal passage	N.D.	[80]
ZnO NPs		*E. coli*		N.D.	[81]
Ag, Pt, NiO, TiO_2_, ZnO NPs		*S. aureus; P. saeruginosa*		N.D.	[82]

Ref., Reference; Ag, silver; AuNCs, gold nanoclusters; CdS, cadmium sulfide; IgG, immunoglobulin; LOD, limit of detection; N.D., not determined; NiO, nickel oxide; NPs, nanoparticles; Pt, platinum; QDs, quantum dots; TiO_2_, titanum dioxide; ZnO, zinc oxide.
